# Evaluating Users’ Experiences of a Child Multimodal Wearable Device: Mixed Methods Approach

**DOI:** 10.2196/49316

**Published:** 2024-02-08

**Authors:** Nancy L McElwain, Meghan C Fisher, Camille Nebeker, Jordan M Bodway, Bashima Islam, Mark Hasegawa-Johnson

**Affiliations:** 1 Department of Human Development and Family Studies University of Illinois Urbana-Champaign Urbana, IL United States; 2 Beckman Institute for Advanced Science & Technology University of Illinois Urbana-Champaign Urbana, IL United States; 3 Herbert Wertheim School of Public Health and Human Longevity Science University of California, San Diego La Jolla, CA United States; 4 Department of Electrical and Computer Engineering Worcester Polytechnic Institute Worcester, MA United States; 5 Department of Electrical and Computer Engineering University of Illinois Urbana-Champaign Urbana, IL United States

**Keywords:** wearable devices, multimodal sensing, user experience, usability, privacy, children, mobile phone

## Abstract

**Background:**

Wearable devices permit the continuous, unobtrusive collection of data from children in their natural environments and can transform our understanding of child development. Although the use of wearable devices has begun to emerge in research involving children, few studies have considered families’ experiences and perspectives of participating in research of this kind.

**Objective:**

Through a mixed methods approach, we assessed parents’ and children’s experiences of using a new wearable device in the home environment. The wearable device was designed specifically for use with infants and young children, and it integrates audio, electrocardiogram, and motion sensors.

**Methods:**

In study 1, semistructured phone interviews were conducted with 42 parents of children aged 1 month to 9.5 years who completed 2 day-long recordings using the device, which the children wore on a specially designed shirt. In study 2, a total of 110 parents of children aged 2 months to 5.5 years responded to a questionnaire assessing their experience of completing 3 day-long device recordings in the home. Guided by the Digital Health Checklist, we assessed parental responses from both studies in relation to the following three key domains: (1) access and usability, (2) privacy, and (3) risks and benefits.

**Results:**

In study 1, most parents viewed the device as easy to use and safe and remote visits as convenient. Parents’ views on privacy related to the audio recordings were more varied. The use of machine learning algorithms (vs human annotators) in the analysis of the audio data, the ability to stop recordings at any time, and the view that the recordings reflected ordinary family life were some reasons cited by parents who expressed minimal, if any, privacy concerns. Varied risks and benefits were also reported, including perceived child comfort or discomfort, the need to adjust routines to accommodate the study, the understanding gained from the study procedures, and the parent’s and child’s enjoyment of study participation. In study 2, parents’ ratings on 5 close-ended items yielded a similar pattern of findings. Compared with a “neutral” rating, parents were significantly more likely to agree that (1) device instructions were helpful and clear (*t*_109_=−45.98; *P*<.001), (2) they felt comfortable putting the device on their child (*t*_109_=−22.22; *P*<.001), and (3) they felt their child was safe while wearing the device (*t*_109_=−34.48; *P*<.001). They were also less likely to worry about the audio recordings gathered by the device (*t*_108_=6.14; *P*<.001), whereas parents’ rating of the burden of the study procedures did not differ significantly from a “neutral” rating (*t*_109_=−0.16; *P*=.87).

**Conclusions:**

On the basis of parents’ feedback, several concrete changes can be implemented to improve this new wearable platform and, ultimately, parents’ and children’s experiences of using child wearable devices in the home setting.

## Introduction

### Background

Advances in pervasive sensing, internet of medical things, and digital health strategies more broadly [1-6] have rapidly accelerated over the past decade. Although digital health research among adults and adolescents has predominantly used smartphones [7-9], parallel work with infants and children tends to use wearable devices [10], including motion sensors to detect body posture and physical activity [11], audio recorders to assess language environment and development [12,13], heart rate sensors to assess psychophysiology [14], and head-mounted cameras to capture infants’ visual perspective of the physical and social environment [15]. Such wearable technology, especially when paired with machine learning algorithms, permits the automated detection of children’s behavioral and physiological states, as well as caregivers’ responses, and has the potential to transform the field of child development through the collection of big data in real-world environments [16].

At the same time, the use of wearable devices among infants and young children in home environments raises unique ethical, legal, and social implications and logistical challenges. As such, careful attention to the perspectives and experiences of end users of such technology, in this case, parents and their children, is required. In this study, we assessed parents’ perceptions of and experiences with a novel wearable device, LittleBeats, developed specifically for use with infants and young children. Little Beats, which is not Food and Drug Administration approved and used only for research purposes, integrates a microphone, a 3-lead electrocardiogram (ECG) sensor, and an inertial motion sensor to synchronously collect information about infant vocalizations, cardiac physiology (heart rate and respiratory sinus arrhythmia), and motion (eg, physical activity level, position, and discrete movements). The electronics are housed in a 3D-printed case (55×57×13 mm), which is placed on a specially designed shirt that the child wears. Data can be collected throughout the day at home, without the researchers present. In prior papers, we reported on machine learning algorithms used to detect and classify child and parent vocalizations using audio data from the LittleBeats device [17] and child sleep states using all 3 sensor modalities [18]. We also conducted technical validation studies to assess the signal quality of each sensor modality in relation to established laboratory protocols and gold-standard equipment [19]. Complementing these prior reports, we focus here on the critical issue of “user experience” among families and their children aged 1 month to 9.5 years. Using semistructured interviews and parent questionnaires to assess parents’ experiences and perceptions, our mixed methods investigation examined usability, privacy, and perceived risks and benefits.

### The “Digital Health Checklist” for Use in Child Development Research

**Figure 1:**
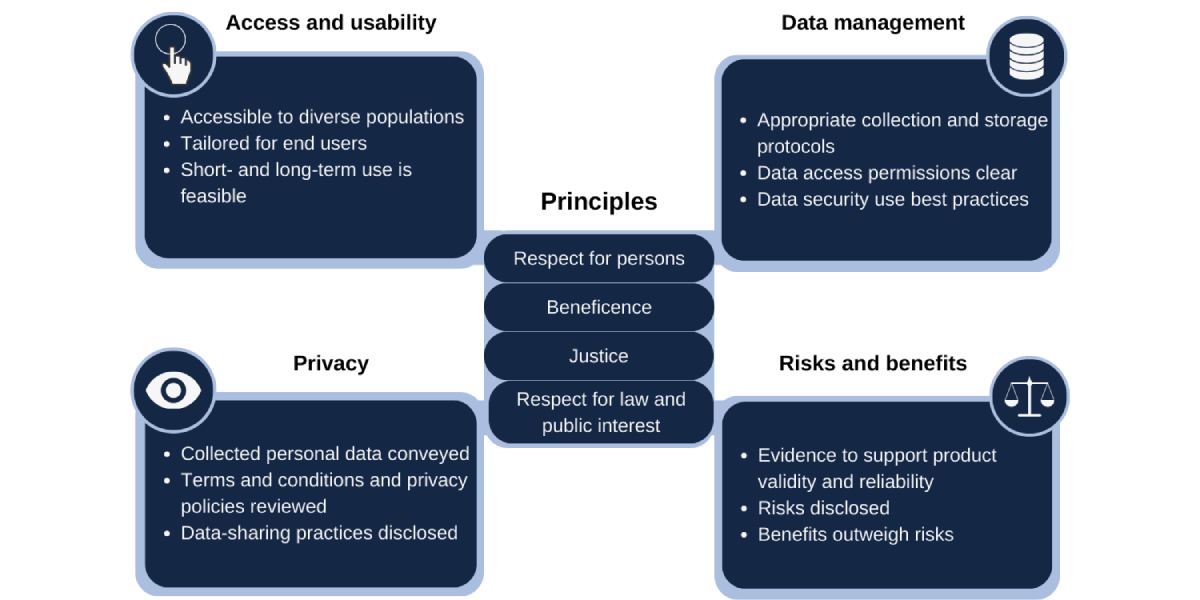
Four-domain framework of the Digital Health Checklist for researchers. The Digital Health Checklist for researchers depicts the 4 ethical principles undergirding the 4 key domains of access and usability, privacy, risks and benefits, and data management. Source: this figure is published with permission and reflects an adaptation of the Digital Health Checklist Developed for Researchers (DHC-R) [34,35].

The proliferation of digital health technologies has spurred a parallel examination of ethical practices and related decision-making processes around the use of such technologies with human participants. To evaluate the LittleBeats platform, we used the Digital Health Checklist developed by Nebeker et al [20,21]; it is grounded in the ethical principles of the *Belmont Report* [22], which speaks to beneficence, respect for persons (or autonomy), and justice, and the *Menlo Report* [23], which added the principle of respect for law and public trust. These principles form the foundation of a 4-domain framework that includes privacy, access and usability, data management (eg, collection, storage, interoperability, and sharing), and assessment of risks and benefits (Figure 1).

To date, the research and development of the Digital Health Checklist has been applied to digital health protocols in adult samples, including for use in cardiovascular disease prevention [24]; studies of human emotion [25]; and improvement of informed consent communications [26]. The current investigation extended the use of the Digital Health Checklist to research involving parents of infants and children. In doing so, we integrated ethical considerations specific to research with children [27]. Specifically, children are a heterogeneous group, and the potential benefits and risks to child participants need to be understood within the context of the child’s age and related physical, cognitive, and socioemotional abilities.

For instance, infants and toddlers may be more susceptible to risks related to emotionally stressful procedures because their coping abilities are less well developed and depend, in part, on support from caregivers. By contrast, older children may be better able to regulate emotions and exert their autonomy, although they might be at an increased risk in other domains. For instance, owing to their growing self-awareness and other awareness, preschool- and school-aged children may be increasingly susceptible to experiencing shame and embarrassment, heightened concerns about privacy, and other related risks to the child’s self-concept.

With developmental differences in risk assessment in mind, we assessed LittleBeats user experience among children representing a large age range (infancy through middle childhood). Although we did not interview children about their study experiences, we considered the children’s age in our analysis of parents’ open-ended responses and parents’ perspectives regarding how their children felt about and responded to the research procedures. Research with children requires parental consent and, depending on the child’s age, the child’s assent to affirm their willingness to participate in the research. The consent process related to the LittleBeats technology has been addressed in a prior report [26]; therefore, we did not consider issues related to the provision of parental consent before participating in this research. Instead, our focus here was on parents’ perceptions of and reflections on their own and their children’s experiences following the use of LittleBeats at home.

Although child development research incorporating the use of wearable devices is rapidly expanding [28-32], systematic assessment of parents’ perspectives and experiences (or ethical considerations more broadly) of such research has been sparse. A notable exception is a report by Levin et al [33], which outlines several key concerns parents may have about participating in research using wearable or remote sensing devices. These concerns focus on privacy expectations, particularly regarding audio or video data (considered “high fidelity data streams”), data management, and data use (eg, for scientific vs commercial purposes). Although we know of no study that assessed parents’ perceptions and experiences of using wearable devices at home *after* data collection, Levin et al [33] provided valuable insights into parents’ general willingness to participate in such research. Among a nationally representative sample of 210 parents (n=105, 50% mothers) with at least 1 child aged ≤5 years, 71.4% (n=150) of parents responding to hypothetical scenarios indicated at least some willingness to participate in studies involving motion or physiological sensors (low fidelity), whereas a significantly lower percentage of parents (n=99, 47.1%) endorsed willingness to participate in studies gathering audio recordings at home. It remains unknown whether the concerns expressed in the study by Levin et al [33], in which parents hypothetically considered participating in different types of remote sensing research, would also be voiced among parents who participated in research in which their children wore a wearable device with multiple sensor types (eg, motion, physiology, and audio).

### This Study

Guided by the domains of the Digital Health Checklist [20], we assessed parents’ experiences with and perceptions of using LittleBeats at home using a mixed methods approach. In study 1, we conducted a qualitative (thematic) analysis of parental responses to a semistructured interview following the completion of 2 day-long LittleBeats recordings at home; children in this study were aged between 1 month and 9.5 years. In study 2, we collected data on parents’ perspectives of using LittleBeats (again, following the completion of several day-long recordings at home) from a separate, larger sample. In this second study, we administered close-ended questionnaire items developed considering the qualitative themes identified in study 1. The parents in study 2 also had the opportunity to provide open-ended comments. In study 2, we narrowed our developmental focus to children aged 1 month to 5 years because our substantive interests focused on early childhood, and analytic tools are currently being developed for LittleBeats data collected among children aged ≤5 years.

## Study 1

### Methods

#### Participants

A total of 47 families with children aged 1 month to 9.5 years were recruited through web-based forums (eg, Facebook [Meta Platforms, Inc] parenting groups) and flyers distributed to local organizations (eg, libraries and day care centers) in a small Midwestern city. Because the larger study from which data were drawn included assessments of child stress physiology, families were excluded if their children had any known cardiac abnormalities. Of the 47 families that participated in the larger study, 42 (89%) completed the follow-up interview about their experience of using LittleBeats at home. Interviews were not completed with 5 (11%) families because of losing contact with them or because interview procedures were not finalized at the time of their study participation.

From these 42 families, 43 children (n=20, 47% female) participated. In 1 instance, 2 (5%) children (aged 13 and 71 mo) were from the same family. Children were aged 1.1 month to 9.5 years (mean 44.9, SD 38.36 mo) and represented 6 age groups: young infants (aged 1-5 mo; 7/43, 16%), older infants (aged 6-17 mo; 10/43, 23%), toddlers (aged 18-35 mo; 7/43, 16%), preschool-aged children (aged 36-59 mo; 6/43, 14%), early school-aged children (aged 5-7 y; 7/43, 16%), and school-aged children (aged 8-10 y; 6/43, 14%). Overall, 22 (51%) children were first born, 11 (26%) were second born, and 9 (21%) were third or later born. Mothers were aged, on average, 35.04 (SD 4.09) years, and fathers were aged, on average, 37.42 (SD 4.48) years. Across mothers and fathers, the highest level of education reported included a high-school degree (1/79, 1%), some college or 2-year degree (18/79, 23%), a bachelor’s degree (22/79, 28%), or an advanced degree (38/79, 48%). Parents identified as Black (2/79, 3%), Asian (3/79, 4%), White non-Hispanic (70/79, 89%), Hispanic (2/79, 3%), or >1 race (2/79, 3%). These demographic data were missing for 2 (5%) of the 42 mothers and 3 (7%) of the 42 fathers. The mean family income was US $79,500 (SD US $25,000).

#### Ethical Considerations

This study was approved by the institutional review board at the University of Illinois Urbana-Champaign (protocol #21032).

#### Overview of LittleBeats Procedures

**Figure 2:**
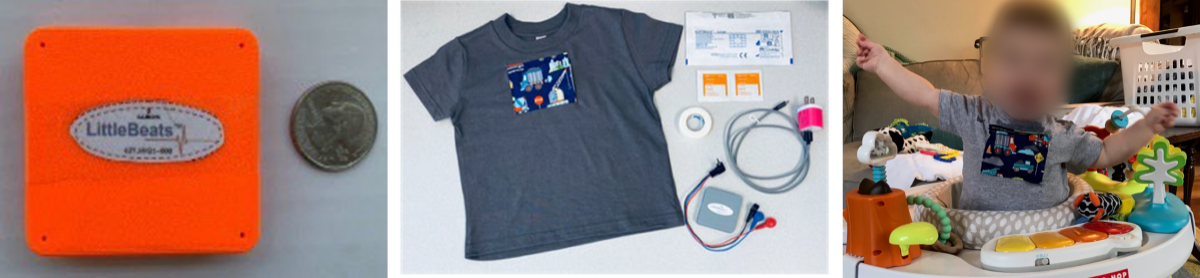
(A) LittleBeats device case; (B) LittleBeats supplies, including electrocardiogram leads, electrodes, charger, and shirt; and (C) an infant wearing LittleBeats at home.

LittleBeats collects 3 streams of data (ECG, motion, and audio data) simultaneously while participants go about their everyday routines (Figure 2). Owing to COVID-19 protocols, all participant engagement was remote. LittleBeats kits (ie, LittleBeats device and shirt, ECG leads, disposable ECG electrodes, alcohol swabs to remove residue from electrodes, medical tape to secure wires on the child’s chest, charging cable and block, and setup instruction cards) were either mailed or delivered by a research coordinator to the family’s home. After receiving the kit, the mother and child met with the study coordinator through Zoom, a secure video web-conferencing platform. During this 40-minute Zoom visit, the study coordinator guided the mother through the LittleBeats setup (described in more detail subsequently), and the mother-child dyad participated in a series of tasks (video recorded for subsequent coding), including a baseline assessment of child stress physiology at rest and a mother-child play session.

For child participants aged <7 years, the mother-child dyads were also asked to complete a brief series of age-appropriate motion interaction tasks, such as the mother picking up her child (aged 1-4 mo), the mother and child (aged 11 mo) clapping together, or the mother and child (aged 6 y) playing “Simon Says.” Toward the end of the Zoom visit, the study coordinator provided instructions for completing the LittleBeats home recordings. Families were asked to complete 2 day-long recordings (approximately 8 hours per day). All adults present at home during the recordings (eg, parents, grandparents, and babysitters) were required to provide consent to the LittleBeats recordings using a secure web-based form provided by the research team. If any nonconsenting adults were at home, parents were asked to turn off the device while these individuals were present. At the end of each day of recording, parents (usually mothers) completed a brief questionnaire about the day’s recording (eg, recording start and stop times). To compensate the families for their time, parents were sent a US $100 e-gift card.

With regard to setting up LittleBeats, the research coordinator walked the mother through the following setup steps at the beginning of the Zoom visit: (1) threading a set of ECG lead wires (20 cm) through the back of the shirt pocket, (2) connecting ECG leads jack (2.5 mm) to the LittleBeats device, (3) turning the device on by sliding the switch to the “on” position (confirmation that the beginning of the recording is indicated by a red flashing light displayed on the device), (4) placing the device in a snug, specially designed shirt pocket, which is secured using 2 snaps, (5) snapping leads to 3 repositionable latex-free gel electrodes, (6) putting the LittleBeats shirt on the child, (7) cleaning the skin (where the electrodes will be placed) with an alcohol prep pad and then placing the electrodes on the child’s skin, and (8) applying a small strip of 3M Micropore medical tape to each ECG wire approximately 5.1 cm below each ECG sticker to help secure the wires in place.

At the end of the Zoom visit, the research coordinator also walked the mother through how the LittleBeats device should be removed. The removal steps include (1) removing electrodes from the child’s skin and using provided alcohol wipes, as needed, to remove residual gel from the electrodes; (2) unsnapping the electrodes from the ECG wires; (3) taking off the LittleBeats shirt; (4) removing the device from the shirt pocket; (5) sliding the slide switch to the “off” position; and (6) plugging the device into the provided charging cable (microUSB cable).

#### LittleBeats Device Design and Study Implementation for End Users

LittleBeats was developed with parents and children (ie, end users) in mind. To provide a context for parents’ interview responses about their study experiences, we noted several aspects of the device design and study implementation intended to proactively increase usability and decrease concerns about privacy. With respect to usability, we provided participants with clear, illustrated instructions in several formats (eg, hard copy and on the web). The device was also designed to be simple to use, with an on-off switch and a charging port, and we provided parents with the all the materials in the LittleBeats kit (refer to the *Overview of LittleBeats Procedures* section) that they would need to set up and use LittleBeats at home. For the child’s comfort, the device is compact (55×57×14 mm) and lightweight (1.48 oz), with foam padding lining the inside of the shirt pocket in which the device is to be placed. The shirts are adorned with a variety of pocket designs (eg, hearts, animals, trucks, and dinosaurs) to appeal to toddlers and preschool-aged children, and, as part of the LittleBeats kit, families received 2 shirts with different designs. For older children, we provided more age-appropriate solid shirt pockets.

With respect to privacy, audio recordings provide high-fidelity information regarding participants’ lives and require special considerations related to participant privacy, data confidentiality, and recording bystanders (for review, refer to the study by Cychosz et al [13]). Our approach to protecting participant privacy aligns with user-centered privacy protections recommended for mobile health research [36] and a “rights-based” approach adopted increasingly in the United States and used by the European Union (ie, General Data Protection Regulation), according to which individuals have the right to control their personal data, including but not limited to consent, erasure, secure data management practices, and transparency. For example, an important strategy to minimize privacy risks includes giving participants control over recordings [37,38]. In this vein, parents were told at several points during their participation (eg, consent process and consent form, verbally during the Zoom visit, and written instruction card) that they were free to turn off or pause the device at any time and that they could request that their recordings be partially or fully destroyed and not be used in the research. With respect to third-party individuals, parents were also instructed to use the device at home when only immediate family members or other consenting adults are present. Parents were informed that all data files were marked only by identification numbers, machine learning algorithms would be used to process the audio data, research personnel would listen to only snippets of the audio files as part of checks on algorithm development and accuracy, and research personnel were trained to protect participant privacy and would immediately cease listening to audio snippets in instances where personal information (discussion of medical, financial, or other personal issues) is being relayed. To minimize the risk of data being intercepted during transfer (ie, uploading data via wireless or Bluetooth networks), data were stored directly on a microSD card on the physical device, and files were configured in such a way that only study personnel could access the data in a human-readable format (eg, wav files for audio) using a data processing pipeline developed specifically for LittleBeats. Because LittleBeats is not a commercial device, simple modifications can be made to the device firmware (eg, “turning off” ≥1 of the sensors) to suit research goals (refer to the study by Islam et al [19] for details about technical specifications).

#### Parent Interview and Coding Procedures

Upon the completion of the LittleBeats recordings, parents (41 mothers and 1 father) completed a brief phone interview about their experiences of using LittleBeats in the home. To help minimize social desirability biases in parental responses, such as parents’ reports of positive experiences with LittleBeats instructions received during the Zoom visit, these interviews were conducted by a second study coordinator who was not present during the Zoom visit. Guided by the dimensions outlined in the Digital Health Checklist [20], as well as special considerations related to research with children [27], our semistructured interview was designed for the purpose of this research to capture information about parents’ experiences and perspectives regarding access, usability, privacy concerns, and risks and benefits with respect to the use of the LittleBeats device and the process of carrying out home recordings. Participants rarely provided information specific to the fourth domain of the Digital Health Checklist, data management, which encompasses how data are collected, stored, and shared and the extent to which the data are accessible to other systems or interoperability. Given the nature of the LittleBeats data (ie, they are not shared outside the research team, not accessible or integrated with other systems, and not transferred via a wireless or Bluetooth network that might be susceptible to security breaches), the data management theme is somewhat less relevant to LittleBeats than to health applications that might be accessed by multiple users (eg, patients, health care providers, and insurance providers). When parents expressed their views on the processes of data collection, storage, and security in the interviews, they almost exclusively focused on the audio recordings and privacy considerations. Therefore, we coded these responses under the privacy domain.

The interview included 11 open-ended questions, and the study coordinator conducting the interviews used standard probes to gain more insight into parents’ experiences, perceptions, concerns, and questions (Multimedia Appendix 1). The interview questions allowed for feedback from all family members’ perspectives (ie, the participating child, participating parents, and any other children or adults in the home). All parent interviews, conducted by the same study coordinator to ensure consistency, were audio recorded with the participant’s permission. Interview recordings were manually transcribed, and identifiable information (eg, names and birth dates) and conversational placeholders (eg, “uh-huh”) were omitted from the transcripts.

We used Taguette [39], an open-source web-based tool for coding textual qualitative data, to capture prevalent themes in our interview data and followed the 6-step approach to thematic analysis defined by Braun and Clarke [40]. At step 1, a review of the transcripts provided preliminary ideas for codes. At step 2, initial codes were generated based on the data from 5 interview transcripts of parents with children from different age groups. Through a series of team discussions, we developed an initial codebook focusing on areas that fell into the larger categories outlined in the Digital Health Checklist [20]. Three transcripts were then used for training purposes, and 3 researchers individually coded the transcripts. Discrepancies were discussed, and additional changes were made to the codebook. Upon the completion of the training, 1 researcher (who was not informed of the specific study objectives) coded all the transcripts using the refined codebook. Reliability was assessed by having the fourth author code 8 randomly chosen transcripts, and among the parent responses that both coders deemed codable, agreement was excellent (Cohen κ=0.967). At step 3, the research team met on a regular basis throughout the coding process to identify and discuss potential themes. At step 4 and after the completion of coding, final themes were reviewed by checking themes in relation to the entire data set to ensure an accurate representation of the data. At step 5, themes were refined and finalized by providing descriptive labels and definitions. At the final step, we organized the results based on the key domains of the Digital Health Checklist and created a summary table of themes with selected interview excerpts to illustrate the findings.

### Results

#### Overview

Themes identified under the major categories of access and usability, privacy, and risks and benefits are summarized in the subsequent sections. Overall, similar themes were identified across developmental periods, although specific examples illustrating a given theme often differed depending on whether the parent reported on their infant, toddler, preschool-aged child, or school-aged child.

#### Access and Usability

According to the Digital Health Checklist, the domain of access and usability prompts researchers to consider whether the participant will be able to use the device as intended. This may involve evaluating whether the product has infrastructure requirements, such as internet access, as well as whether the device has been successfully used in the target population. In this study, usability refers to parents knowing how and being able to successfully use the LittleBeats device and materials (eg, ECG leads). Furthermore, usability encompasses families’ experience of and ability to adhere to the study procedures more generally (ie, participant burden, eg, completing multiple day-long recordings), beyond the use of the device itself (Table 1).

A majority of parents expressed sentiments regarding their ability to easily operate the device (ie, turning the device on-off and charging the device). Some parents indicated feeling comfortable given their previous experience with comparable equipment, yet other parents with no such prior experience expressed similar views about the ease of use. Parents also commented that the instructions were helpful and appreciated having a variety of resources to refer to, if needed (eg, written instruction card, website, and study personnel contact). Aside from operating the device itself, parents had varying views on the materials needed to place the device on their child. Some parents noted that the design was well thought out and that setting up the electrodes was not complicated. However, other parents indicated some challenges with the materials, such as with threading the electrodes through the back of the shirt pocket.

Parents also expressed differing perspectives about the ease of setting up (and removing) the device. Although many parents felt comfortable placing LittleBeats on their children, some parents noted that gaining their children’s cooperation was sometimes a challenge. For instance, some parents reported difficulty putting the device on their “wiggly, squiggly” infants. Other parents reported reluctance on the part of their toddlers or preschool-aged children, who could express their opinions and desires verbally. Typically, if challenges related to child cooperation were experienced, it was during the setup phase, and parents suggested that once their child was wearing LittleBeats, it was quickly forgotten. Parents expressed that the placement of the device on the upper anterior torso (ie, chest) may be disruptive to some activities, such as napping for a child who is a tummy sleeper. Relatedly, the device being concealed in the shirt pocket, with the ECG leads underneath the shirt, was viewed as a disadvantage by some parents who wanted to know whether the device was recording properly or whether there was a malfunction (eg, device turned off or ECG electrodes fell off).

With respect to participant burden, parents expressed a mix of perspectives. Many parents described day-long recordings (ie, >8 h/d) as feasible but challenging. However, parents noted factors that mitigated this challenge, such as the need to record for only a limited number of days spaced across multiple weeks, the ability to schedule their recordings when it worked for them, and the reduction in other competing activities due to the COVID-19 pandemic. In the same vein, parents expressed wanting more features to help them fulfill project expectations. Currently, the device provides no information to the user beyond an indicator light showing that the device is powered on. Parents found it difficult to know how long they had recorded for or how much battery charge was left when using the device.

In addition, many parents described the project as convenient, indicating that the remote data collection procedures were appealing. Being able to collect data at home, on their family’s own schedule, made it relatively easy to participate. Parents were not burdened by the need to travel to a research laboratory, and they could set up the device and start recording when it fit their schedule. Concerns about being able to keep the device on securely or ensure that the device was collecting data were voiced by some parents of older and more active children (eg, increased unsupervised time and gel adhesive weakening owing to perspiration). Other parents expressed their worry that their children would damage the device during data collection.

**Table 1:** Themes, subthemes, and example excerpts related to the access and usability of the LittleBeats device and study procedures (study 1).

Themes and subthemes		Example excerpts
Operating the device		“Everything was pretty easy. It was easy to charge, it was easy to you know put the stickers on and attach, and like I said I don’t think she really felt like it was on. The first day after she asked after an hour ‘how long have I had it on’ I was like ‘why is it uncomfortable’ she was like ‘no I was just wondering’ and I was like ‘oh okay.’ I don’t think she even realized she had it on half the time.” (Parent of a school-aged child)
Instructions		“They [the instructions] were very clear. I mean they made it so that I felt confident putting it on her and doing what I was supposed to do.” (Parent of an older infant)
**Support materials (specially designed t-shirt, wires, and electrodes)**		
	Ease of use	“I think the t-shirt definitely made it easier to use. That was a nice little set up, and it made it, you know, stay in place and like see where it [the device] needed to be for it to be hooked up and stay in place...And then even with the hole on the inside [of the shirt] to make it easy to get all the cords. That was really a unique design tool but effective.” (Parent of a school-aged child)
	Challenges	“It was a little hard getting the black metal piece through the back of the shirt. Like I needed that hole to be a little bigger. So, I’m sure I ripped mine just a little bit...But I just made it a little bit looser.” (Parent of an older infant)
**Setting up and removal of LittleBeats**		
	Comfort with set up	“I’m pretty comfortable getting it set up and turning it on. It seems pretty straightforward.” (Parent of a school-aged child)
	Child cooperation	“It was mostly the initial putting it on. She didn’t want to cooperate with letting us get it on...but after a little bit she forgot it was there because she didn’t have any issues messing with it and then when it was time to take it off she was fine.” (Parent of a preschool-aged child)
	Location of device	“I wish the device itself was a little more discreet. Well, he’s a stomach sleeper so for naps I had to take it off but if it was a little more discreet or was not in front of the t-shirt but maybe on the arm it would be more convenient.” (Parent of an older infant)
**Participant burden**		
	Time commitment	“You know once we broke it up a little bit we could [complete recordings]. I was more worried about you know were rarely all home just the four of us especially now that quarantine is over...We’re just more on the go than we were a year ago.” (Parent of an early school–aged child)
	Convenience	“It was really easy for me as a parent. I drive my other son like I said to [research lab in different city] a bunch...and so that is just a drag, a lot of back and forth. But for I would say from a parent’s standpoint, this was very easy for me to do.” (Parent of an early school–aged child)
	Worry about recordings	“My son’s pretty active, so he sweats a lot over the course of the day. The little stickers would kind of migrate a little bit...So, I worry a little bit that the first recording like the second half of the day might not be as accurate as it was supposed to be.” (Parent of a preschool-aged child) “It would be nice if there were some kind of indicator of battery more visible. And it was also, you know, since I had to take it on and off then count the time, that was also kind of challenging...so some kind of indication of time would also be awesome but I don’t know how complicated it would be to make it.” (Parent of an older infant)
	Worry about device	“A lot of the activities that she wants to do involve painting or drinking water...those kinds of worrying me every time she picks them up. I was more concerned about the hardware.” (Parent of a preschool-aged child)

#### Privacy

The privacy domain focuses on the types of personal information that are or will be collected about participants. In this study, privacy relates to participants’ expectations about and understanding of the process of data collection, in general, and the audio recordings, specifically. Furthermore, this category encompasses the control that participants had over the data collected (Table 2).

Many parents commented that they were initially apprehensive about the home audio recordings but that their worries subsided when provided with more details during the initial informational call with the study coordinator. Other parents noted feeling more comfortable with the audio recordings over time as they participated in the study. Some parents discussed that although they had no concerns, their spouse or partner did. Typically, only 1 parent (usually the mother) was present for the initial informational call with the study coordinator, and this parent then conveyed information to the other parent, which often sufficed to relieve privacy concerns.

By contrast, for some parents, positive views of research, such as having trust or placing value in research, negated concerns about privacy. Other participants described not being concerned with the audio recordings because they “had nothing to hide.” From this view, the audio would capture a typical day in their life, and participants elaborated by describing that the recordings would include everyday family discussions as well as arguments, which participants conveyed as just part of ordinary family life. Others’ lack of concern regarding the audio recordings stemmed from their ability to control when they were recording and, consequently, what was being recorded. They described the process of turning the device on and off as relatively easy and, therefore, reported turning the device off when they were discussing private matters. Some participants mentioned developing ground rules ahead of time to ensure that private information was not discussed when recordings were taking place and, if needed, would alert or remind other family members of the recordings.

The possibility of recording other individuals beyond immediate family members was considered. In working to respect others’ privacy, the participants mentioned several challenges. Some participants expressed that they altered their typical day to avoid interacting with others so that they would not have to worry about unintentionally recording a nonconsenting individual. Other participants stated that although they had planned to record at convenient times when no nonconsenting individuals were around, unexpected situations arose. In addition, although parents had the ability to control when the device recorded, some parents acknowledged that remembering to turn off the device when others were around could be challenging.

**Table 2:** Themes, subthemes, and example excerpts related to privacy concerns about the LittleBeats audio recordings (study 1).

Themes and subthemes			Example excerpts
Initial apprehension about audio recordings			“Cause that was my husband’s big question like ‘are they just going to sit and listen to our day?’ So, he was a little worried about that but once it was explained [that machine learning algorithms would be used to analyze the audio data] he was more comfortable and on board.” (Parent of an early school–aged child)
**Unconcerned about audio recordings**			
	Former views or experiences of research	“She [study coordinator] also told me that it is only used for research purpose and nothing else...I actually love to participate [in research studies]. It is only used for research purposes, so that’s okay.”(Parent of a preschool-aged child) “I was in a study when I was pregnant and we did something similar...my understanding was that the recordings just gets run through the software so we really don’t have anything any interesting happening here so I wasn’t terribly concerned about that [the audio recordings].” (Parent of an older infant)	
	Just an ordinary family context	“I explained everything to everybody [family members, including older children in home]. I do remember there was one particular situation where my 10-year-old was getting into trouble and afterwards he said, ‘Well, they’re gonna hear that!’ And I said this is just a regular family, there’s nothing to be embarrassed about or whatever.” (Parent of a toddler)	
	Ability to control the recordings	“My husband’s a veteran, and he works at the V.A..., so we had to make sure we turned it off before he came home from work because a lot of times he talks about his day.” (Parent of a toddler)	
**Respecting others’ privacy**			
	Adjusting routines or activities to accommodate the study	“I think the only thing is that we didn’t go play with some friends across the street those days where we would’ve otherwise. Like it impeded a little bit of our typical routine, but it felt pretty unobtrusive.” (Parent of a preschool-aged child)	
	Unexpected situations	“When something was happening that I wasn’t expecting, like when I would get a phone call or something like that, and I was just a little concerned about remembering to turn off the device.” (Parent of an older infant)	

#### Risks and Benefits

Evaluating the risks of possible harms in relation to the possible benefits resulting from the knowledge to be gained from the research is linked to the principle of beneficence. Study benefits should outweigh the possible harm to participants and the groups they represent. Risk assessment includes evaluating the type of harm, psychological, physical, reputational, or economic. In addition, researchers must consider the duration, severity, and intensity of the possible harm. Specific to the risks associated with the use of LittleBeats at home, parents expressed varying views along several dimensions, including safety, child comfort, and understanding of the research and its direct outcomes for participants (Table 3).

Many parents expressed that they thought the device was safe for their children to wear. These parents described not being concerned about safety because of the design of the device and the protective features built into it (eg, device was enclosed, tape-covered wires, fitted shirt, and pocket with secure snaps). Some parents indicated that they initially had safety concerns (eg, the device being close to the skin and use of Bluetooth to transfer data) before learning more about the device and its setup (eg, the device itself is not in contact with the skin but is placed in a padded pocket, data are stored directly on the device, and Bluetooth is not used for data transfer). In some instances, parents detailed concerns about their children wearing the device in unsupervised contexts, such as during naptime, and they preemptively removed the device before naps.

Parents also commented on their children’s level of comfort or discomfort. Several parents mentioned that they observed their child functioning normally, such as engaging in typical routines and activities. Parents also stated that their children did not express any discomfort and did not seem to notice that they were wearing the device after a while. Other parents noted their children’s discomfort in putting on or removing the electrodes and medical tape used to secure the wires on the chest. Some parents worried about how comfortable it would be if the child were to hit the device on another object, such as the edge of a table.

Finally, parents’ understanding of the research and its direct outcomes for their families may confer risks and benefits. Some parents revealed a limited understanding of how the data would be used (ie, the ultimate outcome of the research process) or wanted direct feedback on their children’s development, which could pose unintended risks (eg, unfulfilled expectations of direct benefits). Other parents voiced the benefits attributed to participating in the research project itself. For instance, participation provided dedicated time spent together as a family, or completing the surveys was an opportunity to reflect on their children’s activities and development. Several parents expressed their desire to contribute to the project because they recognized the importance of the research. Some parents indicated that they had enjoyed participating in previous studies, and others stated that this project’s description seemed interesting and fun. Other parents of older children revealed that when they initially talked to their children about the study, their children seemed interested in participating, so they signed up. Some participants communicated that their children enjoyed participating in the project, with one parent acknowledging that their children felt special for a day while wearing the LittleBeats shirt.

**Table 3:** Themes, subthemes, and example excerpts related to the risks and benefits of participating in the LittleBeats study (study 1).

Themes and subthemes			Example excerpts
Safety			“No [safety concerns] because all of the wires were covered by her shirt and taped down.” (Parent of an older infant) “Not really [any safety concerns]. I mean the wires were short enough that I wasn’t worried about them.” (Parent of an early school–aged child) “I thought it will get like hot because I recorded for the 8 hours straight, I didn’t stop it at all, I was worried maybe it’s gonna be hot or something, but it wasn’t hot at all. That was my main concern only.” (Parent of a younger infant) “And then I did have an initial concern...about the safety of having that device running on Bluetooth. I’m not sure how it communicates data and that being so close to skin.” (Parent of an older infant)
Child’s comfort or discomfort			“I guess putting them [electrocardiogram electrodes] on wasn’t the hard part. The hard part was taking them off, especially the was a little bit hard, and my son is also not very fond of changing clothes.” (Parent of an older infant) “I’d probably take it off especially because my little one is about 10 1/2 months and she’s a tummy sleeper so that would be uncomfortable.” (Parent of a preschool-aged child) “I mean it seemed it was fine. My sons were playing outside you know riding their bikes and everything and they didn’t...say anything was uncomfortable.” (Parent of an early school–aged child)
**Outcomes of participating in the research**			
	Limited understanding	“I would love to know what kind of information. I know what kind of information they collected with the device and I’m just curious what they are going to use it for in the future.” (Parent of an older infant)	
	Understanding gained	“[Filling out] this survey, I found that I am pretty lucky that my son is more adaptable. The question, was for example, ‘when you want him to go to bed, he just cried or tantrum’ but he never does that.” (Parent of a preschool-age child)	
	Parent’s enjoyment or satisfaction	“I just like participating in research and helping out the scholars. In my undergrad, I was doing some research and I know how important it is and how hard it can be so...I think it’s good to help.” (Parent of an older infant) “I actually like to spend time with my son. He goes to school every day, so I like to do something with him like the zoom interview. And also I want to show him new technologies.” (Parent of a preschool-aged child)	
	Child’s enjoyment	“I didn’t mind the surveys or anything, and my son loved wearing the LittleBeats. He kept asking if he could put them on. So, I think it captured the kid’s interest too.” (Parent of an early school–aged child) “We had fun doing it [the study], and I think [my son] enjoyed being special, wearing his special shirt for a day.” (Parent of a toddler)	

## Study 2

### Overview

Building on the key themes of access and usability, privacy, and risks and benefits identified in study 1, we administered a brief survey among a larger sample of parents participating in a different LittleBeats study with children aged 0 to 5 years. Although our main interest was to complement the qualitative findings of study 1 with a quantitative assessment of parents’ perceptions using close-ended rating scales, parents were also able to provide open-ended comments. Therefore, we have also summarized the main themes reflected in these open-ended comments.

### Methods

#### Participants

In study 2, a total of 110 parents (n=108, 98% mothers and n=2, 2% fathers) completed a user experience survey after completing 3 days of LittleBeats recordings at home. Recruitment procedures were similar to those described in study 1. Children (60/110, 54.5% female) were aged, on average, 23.4 months (SD 16.87 mo; range: 2-65 mo) and were identified by parents as Black (n=5, 4.7%), Asian (n=8, 7.5%), White non-Hispanic (n=67, 63.2%), Hispanic (n=15, 14.2%), or >1 race (n=11, 10.4%). Children were first born (n=50, 47%), second born (n=39, 38%), and third or later born (n=17, 15%). Parents were aged, on average, 34.85 (SD 5.01) years, and their highest level of education reported included some high school or high-school degree (4/106, 3.8%), some college or 2-year degree (9/106, 8.5%), a bachelor’s degree (33/106, 31.1%), or an advanced degree (60/106, 56.6%). Parents identified as Black (7/106, 6.6%), Asian (13/106, 12.3%), White non-Hispanic (75/106, 70.8%), Hispanic (8/106, 7.5%), or >1 race (3/106, 2.8%). The mean family income was US $83,250 (SD US $26,470). Of the 110 parents, 4 (4%) were missing responses on the demographic survey but did complete the LittleBeats user experience survey described subsequently.

#### Ethical Considerations

This study was approved by the institutional review board at the UIUC (protocol #22631).

#### Procedure

Families were mailed a LittleBeats kit and participated in a Zoom visit, during which a study coordinator walked the parent through the LittleBeats setup and a visit procedure consisting of a baseline assessment of child stress physiology and parent-child interaction tasks (eg, play). At the end of the visit, parents received instructions about completing the day-long recordings and were asked to complete 3 day-long recordings over the course of 2 weeks. Parents also completed a series of web-based questionnaires about family demographics, child behavior, and family functioning. Parent questionnaires were administered either via Qualtrics or REDCap (Research Electronic Data Capture; Vanderbilt University [41,42]) hosted at the UIUC, with the support of the Interdisciplinary Health Sciences Institute and Research IT—Technology Services at the UIUC. Both web-based software platforms are designed to support secure data capture for research studies. Once parents returned the LittleBeats kit by mail, 1 parent in the household (who had been involved in setting up and carrying out the LittleBeats recordings) was asked to rate 5 items about their experience of using LittleBeats, including setting up LittleBeats, along with their perceptions of safety, privacy, and participant burden. Each item was rated on a 5-point scale ranging from 1 (*strongly agree*) to 5 (*strongly disagree*). Following each item, parents had the opportunity to add comments or elaborate on their rating. A final open-ended item also asked parents whether there was anything else they would like to share about their experience or anything they would tell someone who was considering joining a LittleBeats study.

#### Data Analytic Plan

Descriptive statistics, including the frequency distribution, for parental ratings on each of the LittleBeats user experience items were examined. For each close-ended item, we conducted a single-sample *t* test (2-tailed) to determine whether the mean rating significantly differed from the midpoint of the scale (ie, value of 3=“neutral”). Finally, using the coding scheme developed in study 1, we assessed themes from parents’ responses to the open-ended items.

### Results

#### Parents’ Ratings on User Experience Items

Percentage frequency distributions of parents’ ratings on the user experience items are shown in Table 4. Single-sample *t* tests indicated a significant difference between the item average (lower ratings indicated greater agreement; higher rating indicated greater disagreement) and the midpoint of the rating scale (3=“neutral”) for 4 (80%) of the 5 items. Compared with a “neutral” response, parents were significantly more likely to *agree* that (1) the LittleBeats instructions were helpful and clear (mean 1.21, SD 0.41; t_109_=−45.98; *P*<.001), (2) they felt comfortable setting up LittleBeats on their child (mean 1.42, SD 0.75; t_109_=−22.22; *P*<.001), and (3) they felt their child was safe while wearing LittleBeats (mean 1.33, SD 0.51; t_109_=−34.48; *P*<.001). Compared with a “neutral” response, parents were significantly more likely to *disagree* that they worried about being recorded by the LittleBeats device (mean 3.62, SD 1.06; t_108_=6.14; *P*<.001). The final item tapped parents’ perceptions of burden (“I felt that completing LittleBeats recordings for full 3 days was challenging”), and the item average (mean 2.98, SD 1.17) did not significantly differ from “neutral” (t_109_=−.16; *P*=.87).

**Table 4:** Frequency distributions of parental rating of the LittleBeats user experience survey (study 2; n=110).

Survey item	Strongly agree, n (%)	Agree, n (%)	Neutral, n (%)	Disagree, n (%)	Strongly disagree, n (%)
The instructions to setup LittleBeats were helpful and clear.	87 (79.1)	23 (20.9)	0 (0)	0 (0)	0 (0)
I felt comfortable setting up LittleBeats on my child.	75 (68.2)	29 (26.4)	2 (1.8)	3 (2.7)	1 (0.9)
I felt my child was safe while wearing LittleBeats.	76 (69.1)	32 (29.1)	2 (1.8)	0 (0)	0 (0)
I was worried about being recorded when the LittleBeats device was on.	3 (2.7)	13 (11.8)	32 (29.1)	35 (31.8)	26 (23.6)
I felt that completing LittleBeats recordings for 3 full days was challenging.	11 (10)	32 (29.1)	26 (23.6)	30 (27.3)	11 (10)

#### Parents’ Responses to Open-Ended Items

A review of parents’ responses to the optional item to add further comments following each of the rating scales revealed themes that closely mirrored study 1 findings. Regarding the ease-of-use item, 29 (26.4%) of the 110 parents added comments. Most parents noted that having an instruction card included in the kit, as well as a QR code to easily link to the website for more detailed instructions, increased usability.

Regarding comfort in setting up the device, 23 (20.9%) of the 110 parents added comments. Parents noted that they felt comfortable and that the setting up of the device was easy. However, parents also noted that the process of setting up the device was difficult when their child moved around. Other parents mentioned the comfort level of their child (eg, noting that their child felt discomfort when removing the ECG electrodes).

Regarding safety, 25 (22.7%) of the 110 parents added comments. Parents noted few concerns because the device was concealed in a pocket and not easily accessible to the child. Parents who expressed a concern commented on the placement of the device on their child’s chest.

Regarding concerns about being recorded, 32 (29.1%) of the 110 parents added comments. Some parents noted feeling self-conscious about their parenting or other family members’ language choices. Typically, these comments were followed by comments about feeling relieved that the audio would be processed by a machine (vs a human coder). By contrast, many parents explained that they went about their day as usual, which typically contained some sort of sibling argument or other family disagreements.

Regarding participant burden, 68 (61.8%) of the 110 parents added comments. Unlike in study 1, where participants were asked to use the device for 2 days, study 2 participants were asked to use the device for 3 full days (or a total of about 24 hours) over the course of 2 weeks. Several parents commented on their families’ busy schedules and difficulty finding 3 full days when only immediate family members were present.

Finally, a number of parents (46/110, 41.8%) responded to the final open-ended question asking whether they had any other comments they would like to share. Responses mirrored study 1 themes in several respects, including parents’ and children’s enjoyment in participating in the study (eg, “fun and easy” and “I would recommend to my friends”), children’s ability to forget about the device and go about their usual day (eg, “did not interfere with our day”; “[Child] did not notice the device...he was able to nap with it on and so it was really pretty simple to participate!”; and “once the shirt was on, she forgot it was there and so did I!”), and suggestions for ways to minimize burden and improve the experience (eg, adding a display on the device that provides more information about battery charge, power status, and recording length).

## Discussion

### Summary

Digital health technologies have largely been developed with adults in mind. Interest in and attention to the use of wearable devices among infants and young children, however, has been growing, and data collection using wearable devices provides several advantages over traditional data collection methods, including continuous assessment, greater ecological validity, and the automated detection of behaviors using machine learning algorithms. Given these advantages, combined with rapid technological advances, it is likely that the use of wearables in child development research will burgeon in the coming years. Therefore, assessing how such devices and related data collection protocols are perceived and experienced by parents and their children is critical. User experience studies not only address ethical considerations but can also lead to important changes in research protocols that address parents’ concerns and increase the benefits for future families who participate. Indeed, our mixed methods investigation across 2 studies yielded consistent findings that shed light on parents’ experiences and perceptions of LittleBeats’ usability and safety, the privacy of the audio recordings, and potential risks and benefits of participating in research of this kind. A large majority of parents indicated that device instructions were helpful and clear, the device was easy to use and safe, and remote visits were convenient. Parents’ views about privacy, risks, and benefits were more varied, although, on average, parents reported feeling comfortable with the audio recordings. In summarizing the major themes identified within the major categories, we consider ways in which the findings can inform the future design and implementation of wearable platforms in child development research.

### Key Findings

Results across all themes underscored the variability in parents’ (mostly mothers’) perspectives and experiences. With respect to access and usability, some parents expressed interest in having access to information that indicated the cumulative time recorded as well as the battery charge remaining. Such additions to the platform would eliminate parents’ need to track the recording length and minimize parents’ concerns about whether the device was sufficiently charged and recording. Some parents also noted difficulty with threading the ECG lead wires through the back of the shirt or were worried that their child would tug on the wires. These challenges can be remedied by changing the shirt design such that the ECG wires would be more fully integrated into the shirt fabric or design. Although parents indicated that day-long recordings (ie, >8 h/d) were feasible, some parents noted challenges. To alleviate the burden of day-long recordings, the time requirements can be adjusted to be more flexible. For instance, parents can be asked to complete recordings for fewer hours per day across multiple days (ie, 3 to 4 h/d across 4 to 5 d), although the optimal length and frequency of recordings needed to reliably capture the constructs of interest will vary as a function of the research questions being addressed. Importantly, such burdens were balanced by parents’ comments regarding the convenience of remote visit procedures and the ease of using LittleBeats.

Privacy was a theme that also garnered a variety of responses. Some parents indicated few concerns about the privacy of the home audio recordings, whereas other parents worried that the recordings captured private conversations. In the latter case, some families used rules or reminders to control or limit when audio recordings were collected. It is also notable that parents within the same family sometimes expressed differing levels of comfort or concern with the audio recordings. When this pattern emerged, it was largely fathers who voiced concern about invasion of privacy, perhaps because they were not present for initial conversations with the study coordinator, who detailed how the data would be collected and used.

We consider 2 main ways to address parents’ privacy concerns about the home audio recordings (also refer to the study by Cychosz et al [13]). First, providing specific and concrete examples of how the audio recordings are processed and analyzed, perhaps by illustrating a hypothetical example of the data collection, processing, and analysis steps, may help ease privacy concerns. Indeed, some parents noted that the use of machine learning algorithms to analyze the data alleviated their concerns about the audio recordings and privacy-related issues. Thus, describing the machine learning algorithms in a detailed yet accessible manner for nontechnical users and stating ways in which the data will not be used or analyzed (eg, no transcriptions of speech) may help reassure parents. Such information should be provided to all family members participating in the home recordings, including older siblings, and should be presented in various formats (eg, brief informational videos, hard copy pamphlets, interactive web page), along with multiple ways to contact study personnel for questions or comments. As part of this solution and building on some parents’ perspectives that the recordings were just capturing “typical family life,” researchers conducting day-long recordings may also explicitly highlight the family as an important context for development, coupled with appreciation for the fact that all families are different, and that, as researchers, we want to capture what life is like for each family and infant.

A second solution to alleviate parents’ concerns about privacy could involve technological innovations, such as collecting audio recordings in which speech content is not intelligible (refer to the study by Levin et al [33]) or data processing (eg, machine learning algorithms) that occurs on the device or hub in the home so that the audio recordings are not stored or released to the researcher. However, these solutions require further technological advances in audio signal processing and raise issues regarding data-quality assurance. That is, without high-fidelity recordings, the validation and quality checks of machine learning algorithms become difficult. Furthermore, when parents were presented with several hypothetical scenarios for collecting child sensor data in the home environment, parent-reported willingness to participate did not significantly differ between study scenarios in which lower resolution audio data were collected (eg, recording 1-min snippets every 20 min and processing audio data automatically so that raw audio data are not stored) and study scenarios in which higher resolution data (eg, continuous audio recordings) were collected [33]. Taken together, although technological solutions aimed at increasing privacy protection seem to be a reasonable avenue to pursue, future studies on users’ experiences of child wearables, particularly home audio or video recordings, should systematically assess parents’ concerns, needs, and desires when it comes to balancing the privacy of day-long home recordings with the benefits of participation.

Third-party or bystander privacy is also a complex issue [37,38]. In this study, there were two categories of potential third parties: (1) nonparental caregivers or relatives at home who were part of the child’s regular routine and (2) individuals who were not part of the home environment (eg, delivery persons and neighbors). In the first case, nonparental caregivers can be included in the recording if they provided consent. In the second case, the parent would need to turn off the device while the individual is present or change their routine to avoid third parties, which may have consequences for ecological validity. Concerns about third-party recordings can also be resolved by the same types of technical solutions outlined earlier.

The principle of beneficence yielded a variety of responses regarding the risks and benefits of the study procedures. First and foremost, safety was a key theme, and across both samples, parents predominantly expressed views that LittleBeats was safe. When concerns about safety were mentioned, parents often presented hypothetical concerns (eg, the device being close to the skin, the device radiating heat, and the child accidentally falling on the device; the last scenario is mentioned as a potential risk in the parental consent form), which were usually alleviated once the parent learned more about the study. Some parents also mentioned concerns about the child wearing the device during unsupervised times, such as naps, and removed the device during these times. Because infants and young children are much more likely to take ≥1 naps over the course of the day, this subtheme differed across age groups, with parents of children in younger age groups being more likely to mention device use with respect to nap times. Another set of risks is related to the child’s discomfort, particularly around the application and removal of the ECG electrodes. This potential risk is also mentioned in the parental consent form, and we aimed to ameliorate this risk using latex-free electrodes designed specifically for pediatric populations.

Potential or perceived risks were balanced by parents’ perceived benefits, including increased understanding of their child’s development through the completion of the parent surveys, parents’ satisfaction in contributing to the scientific process, children’s enjoyment of the study procedures (eg, play session with parents), and wearing the novel LittleBeats shirt and device. We note that we did not ask directly about perceived benefits in study 2 close-ended items, although parents in this study did indicate the benefits of participation in the final open-ended question asking whether they had any other comments they would like to share. These responses often paralleled the positive sentiments that study 1 parents expressed. Nevertheless, items that assess the perceived benefits of study participation will be important to include in future studies.

With respect to increasing direct benefits to participants, we gave families personalized books summarizing information that we have collected about their children (eg, height and weight at different ages) in prior studies. Such summaries have been well received and appreciated. Similar types of summaries can be made from data extracted from day-long recordings (eg, frequency and duration of infant babbling or crying). Providing this type of study feedback to parents may also promote effective participant recruitment and retention, particularly among studies that involve high-fidelity data, such as audio recordings. As noted by Levin et al [33], individuals are likely to evaluate intrusiveness and data privacy, on the one hand, and direct benefits to themselves and their children (such as receiving useful, personalized information or feedback from the data collected), on the other hand, when making decisions about whether to participate in such research.

### Study Limitations and Future Directions

We note several limitations of our user experience studies. First, we did not ask our older child participants about their experiences directly, although parents reported on a variety of child experiences, including compliance with putting on the device, excitement in wearing the shirt, feeling special while wearing the shirt, and comfort or discomfort. The device hardware was relatively compact and lightweight, and parents reported that children tended to forget about it once it was on. Nonetheless, these reflections clearly highlight the need to directly assess not only parents’ perspectives but also children’s perspectives. Thus, parental reports of their child’s experiences should be augmented by direct observations of infants and younger children while wearing the device as well as interviews with older children. Second, we tracked parents’ reported experiences based on the child’s developmental stage. Similar themes were found across developmental periods, although specific examples of how themes manifested often differed by the child’s age. However, because the subsamples of children in different age groups were relatively small, future research with larger subsamples is needed to more thoroughly investigate developmental considerations related to user experiences in the context of research using child wearables. However, an age-specific consideration that did clearly emerge relates to daytime sleep. Third, in both samples, parents reported high levels of educational attainment. Future research on parents’ perspectives of using child wearable devices in the home setting should include families with diverse demographic characteristics. Including samples characterized by sociodemographic factors in user experience studies is especially critical for child wearables developed for the purposes of mobile health interventions.

### Conclusions

Wearable sensors designed for and validated with infants and young children present researchers and clinicians with tremendous opportunities to assess developmental processes and outcomes in more ecologically valid and potentially less burdensome ways than laboratory assessments. Furthermore, LittleBeats’ multiple modalities provide especially rich data to assess an array of constructs central to child development researchers and clinicians, including parent-child vocal turn-taking, regulation of stress, sleep-wake cycles, physical activity, and developmental disorders. At the same time, although we have validated LittleBeats sensors and machine learning algorithms to accurately capture some of these key constructs [17-19,43], the degree to which LittleBeats and similar child wearables deliver benefits (eg, high ecological validity and low burden) will largely depend on acceptance by the end users (eg, parents and children), making user experience studies critical to this research space. In short, if the technology is not acceptable to the end user, it is less likely to be adopted and used as intended. The user experience assessment presented in this paper goes hand in hand with technical validations of the device, and both are critical for successful implementation. The current results suggest that parents predominantly view LittleBeats as easy to set up and use at home, although views regarding privacy and burden were more varied. On the basis of parents’ thoughtful and specific feedback, several concrete changes can be implemented to improve the LittleBeats platform and, ultimately, parents’ and children’s experiences.

## Appendix

Multimedia Appendix 1 Parent user experience interview.

## Abbreviations

ECG: electrocardiogram
REDCap: Research Electronic Data Capture
UIUC: University of Illinois Urbana-Champaign
