# Supplementary material for: Evaluating Users’ Experiences of a Child Multimodal Wearable Device: Mixed Methods Approach
Source: JMIR Hum Factors. 2024 Feb 8;11:e49316. doi: 10.2196/49316 (PMC10884896; doi:10.2196/49316)
Supplement: Multimedia Appendix 1 [file humanfactors_v11i1e49316_app1.docx]

**Parent “User Experience” Interview**

We’d like to hear about your experience using LittleBeats while at home. These could be things like how easy or difficult it was to use it, what you and/or your family thought about the project and, any ideas you have that might make participating easier or more interesting for other families with [XX-aged] children. We are curious about what is working and what might need to be changed. Your input is very important to us.

***Interview questions (possible probes inserted in parentheses):***

1. Why did you decide to join the LittleBeats study?
2. Now that you’ve completed the study, how would you describe your overall experience?
3. How helpful [or clear] were the instructions for using LittleBeats at home? (*Probe about specific instruction materials, e.g., verbal instructions at the end of the visit, as well as written manual*: Did you find anything confusing? If yes, what might we do to improve our instructions?)
4. How comfortable were you using LittleBeats on your own? (Was it easy to use? Were you uncertain about how you were using it? Did family members express any concerns about LittleBeats?)
5. What problems or concerns came up when using LittleBeats in your home? (*Probe about specific concerns as appropriate*):
   1. What do you think about the safety of LittleBeats? Any concerns for you or your child?
   2. What about privacy? Did you have any worries about your/your family personal information that might be recorded when using LittleBeats?
   3. Did you or your family worry at all about being recorded when the LittleBeats device was on? Intrusive (vs unobtrusive)?
   4. What kind of tech problems, if any, did you experience (e.g., power/charging)?
   5. What, if any challenges did you encounter specific to child compliance & behavior (i.e., child refusing LittleBeats, fussing while wearing LittleBeats, attempting to take off LittleBeats/ECG stickers)
   6. What do you think we can learn from using technologies like LittleBeats in the home setting?
6. We asked you to use LittleBeats for several hours per day for several days. Our goal was to gather about 20 hours of home recordings. How did that go for you? your family? (*Probe, as needed, about specific concerns regarding easy of completing the requested amount of recording and the burden families felt*: Why do you think you were able to use LittleBeats as often as you did? What do you think might have prevented you from using the device more often?)
7. We also requested that you use LittleBeats while only family members were present or to have “other adults” complete the online consent form if they agreed to be part of the recordings. How did that go for your family? (*Probe, as needed, about what happened when other adults were present or logistical issues in recording when only family members were present*):
   1. Did you need to use the online consent option?
      1. If yes, how did that go for you?
   2. What did your family members think about participating in the LittleBeats study?
8. Do you have any feedback on the brief survey you completed on each day that a LittleBeats recording was made? (*Probe about burden to complete survey; difficulties completing survey due to forgetting, time commitment, etc. or because of how the items were formatted or worded.)*
9. What do you think of the specially designed LittleBeats shirt that your child wore? Was your child comfortable in the LittleBeats shirt? What might we do to improve the shirt (appearance, comfort, etc.) (*Probe about perceptions of quality, size, ease of using pocket for inserting device and threading leads, etc*.)
10. Is there anything else you’d like us to know about your experience? What would you tell someone who was considering joining the LittleBeats Study? Do you have any other questions or thoughts about LittleBeats?
